# P-569. Changes in lipid profile in people living with HIV on stable protease inhibitors (PIs)-based regimens as second-line ART regimens switch to dolutegravir (DTG)-based regimens

**DOI:** 10.1093/ofid/ofae631.767

**Published:** 2025-01-29

**Authors:** Natthapol Kiatkangwanchon, Jiratchaya Sophonphan, Anchalee Avihingsanon, Sivaporn Gatechompol, Opass Putcharoen

**Affiliations:** Chulalongkorn university, Pathumwan, Krung Thep, Thailand; HIV-NAT, Thai Red Cross AIDS Research Centre (TRCARC), Pathumwan, Krung Thep, Thailand; HIV-NAT, Thai Red Cross AIDS Research Centre, Bangkok, Krung Thep, Thailand; HIV-NAT, Thai Red Cross AIDS Research Centre (TRCARC), Pathumwan, Krung Thep, Thailand; Division of Infectious Disease, Department of Medicine, Faculty of Medicine, Chulalongkorn University, Krungthep, Krung Thep, Thailand

## Abstract

**Background:**

Antiretroviral therapy (ART) for human immunodeficiency virus (HIV) infection is associated with metabolic changes, including dyslipidemia and cardiovascular risk. Switching from protease inhibitor (PIs)-based regimens to dolutegravir (DTG)-based regimens offers potential benefits, but its impact on metabolic parameters requires investigation. This study aimed to assess the change in low-density lipoprotein cholesterol (LDL-C) levels, a risk factor for cardiovascular disease, in people living with HIV (PLWH) who switched from stable PIs-based second-line ART regimens to DTG-based regimens.

**Table 1:**
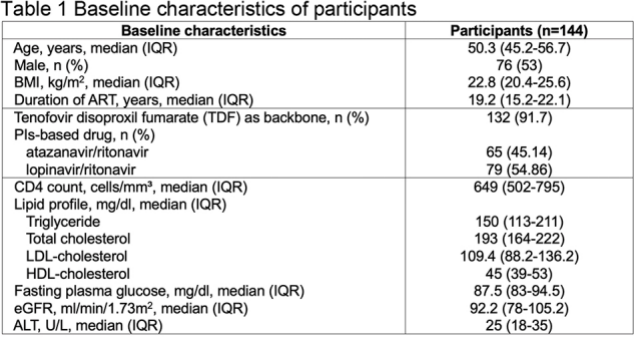
Baseline characteristics of participants

**Methods:**

We conducted a prospective cohort study evaluating lipid profile, renal function, and other metabolic parameters in PLWH who switched from PIs-based to DTG-based regimens. The DTG-based regimens comprised two nucleotide reverse transcriptase inhibitors (NRTIs), specifically tenofovir alafenamide (TAF) and emtricitabine (FTC), along with DTG in a single-tablet regimen once daily. The study was conducted at HIV-NAT, and data were collected over 24 weeks.

**Table 2:**
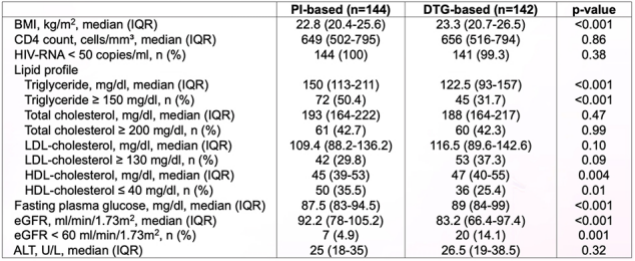
Comparison of BMI and laboratory tests between PIs-based and 24 weeks after switching to DTG-based regimens

**Results:**

Among 144 PLWH participants, switching to DTG-based regimens resulted in significant reductions in triglycerides (TG) and improvements in high-density lipoprotein cholesterol (HDL-C), while LDL-C and total cholesterol (TC) remained stable. Post-switching, participants experienced weight gain and increases in body mass index (BMI). Renal function, assessed by estimated glomerular filtration rate (eGFR), showed a decline. Despite these changes, virological suppression was maintained, and depressive symptoms remained stable. Notably, depression scores assessed using the Patient Health Questionnaire-9 (PHQ-9) at 24 weeks post-switching primarily indicated normal (68.3%) and mild depression (19.8%), with only one participant reporting severe depression.

**Conclusion:**

Switching to DTG-based regimens in PLWH offers benefits in lipid profile and pill burden reduction, with modest increases in weight and LDL-C attributed primarily to the transition from TDF to TAF. Further research is needed to assess long-term effects, particularly on renal function and cost-effectiveness in diverse settings. These findings contribute to optimizing HIV care and treatment strategies globally.

**Disclosures:**

**All Authors**: No reported disclosures

